# A Recombinant Affinity Reagent Specific for a Phosphoepitope of Akt1

**DOI:** 10.3390/ijms19113305

**Published:** 2018-10-24

**Authors:** Jennifer E. McGinnis, Leon A. Venegas, Hector Lopez, Brian K. Kay

**Affiliations:** Department of Biological Sciences, University of Illinois at Chicago, Chicago, IL 60607, USA; mcginn11@uic.edu (J.E.M.); lveneg3@uic.edu (L.A.V.); hlopez2158nct@gmail.com (H.L.)

**Keywords:** affinity selection, alanine-scanning, Forkhead Associated (FHA) domain, phosphopeptide, phage-display, surface plasmon resonance (SPR)

## Abstract

The serine/threonine-protein kinase, Akt1, plays an important part in mammalian cell growth, proliferation, migration and angiogenesis, and becomes activated through phosphorylation. To monitor phosphorylation of threonine 308 in Akt1, we developed a recombinant phosphothreonine-binding domain (pTBD) that is highly selective for the Akt1 phosphopeptide. A phage-display library of variants of the Forkhead-associated 1 (FHA1) domain of yeast Rad53p was screened by affinity selection to the phosphopeptide, 301-KDGATMKpTFCGTPEY-315, and yielded 12 binding clones. The strongest binders have equilibrium dissociation constants of 160–180 nanomolar and are phosphothreonine-specific in binding. The specificity of one Akt1-pTBD was compared to commercially available polyclonal antibodies (pAbs) generated against the same phosphopeptide. The Akt1-pTBD was either equal to or better than three pAbs in detecting the Akt1 pT308 phosphopeptide in ELISAs.

## 1. Introduction

The serine/threonine-protein kinase, Akt1, is responsible for regulating a range of biochemical pathways involved in cell proliferation and survival. It contains an N-terminal pleckstrin homology (PH) domain, a serine/threonine kinase catalytic domain, and a C-terminal regulatory domain [1,2]. Activation of Akt1 is dependent on recruitment of the protein, through its PH domain [3], to the inner side of the plasma membrane, which causes a conformational change [4], allowing PDK1 to phosphorylate threonine 308 (T308) in Akt1’s catalytic domain and mTORC2 to phosphorylate serine 473 (S473) in Akt1’s regulatory domain [5,6]. Once these two residues are phosphorylated, Akt1 is fully active and phosphorylates a range of intracellular proteins involved in cell survival, growth, proliferation, cell migration and angiogenesis [7].

Given the critical roles that Akt1 serves in the cell, biologists are extremely interested in understanding its involvement in cancer. Mass spectrometry and phospho-specific antibodies have been essential tools in pursuing this question by tracking Akt1’s phosphorylation state and levels in cells and tissues [8,9]. Such methods have shown a strong link between the hyperactivation of Akt1 through increased phosphorylation levels in breast, prostate [10], ovarian [11], and pancreatic cancer [12]. Additionally, studying phosphorylation of specific residues within a protein can provide valuable information as some diseases are marked by the excessive phosphorylation of only one or a few of these residues. For example, the phosphorylation of T308, but not of S473, has been characterized as a marker of lung cancer [13]. Thus, antibodies that recognize specific phosphorylated residues as part of their epitopes serve as valuable diagnostic tools to distinguish between diseases caused by Akt1 deregulation.

Unfortunately, mass spectrometry is not well suited to monitoring protein phosphorylation at the cytological level, and antibodies are often poorly validated, unsequenced, and not amenable to protein engineering [14]. To circumvent these limitations, current efforts have been focused on generating engineered protein scaffolds that recognize phosphoepitopes, such as the 10th fibronectin type III domain (10FnIII) [15], designed ankyrin repeat proteins (DARPins) [16], the Src Homology 2 domain (SH2) [17], single chain variable fragments (scFv) [18], antigen binding fragments (Fab) [19], and the Forkhead-associated (FHA) domain [20]. Unlike other scaffolds and most antibodies, FHA domains are selective for phosphothreonine (pT)-containing targets due to a pocket on the domain that interacts with phosphate and γ-methyl group of phosphothreonine (pT) [21,22]. Because of this unique characteristic, a phage library displaying FHA1 variants randomized at residues 82–84 in the β4-β5 loop and residues 133–139 in the β10-β11 loop have been employed to generate affinity reagents to a variety of targets [20,21,23]. Here, we describe the isolation and characterization of Akt1 phosphothreonine 308 (pT308)-binding reagents. We show that these reagents are pT-dependent, bind with high affinity, and recognize the phosphopeptide with comparable or better specificity than commercially made antibodies.

## 2. Results and Discussion

Directed evolution of the FHA1 domain yielded variants that recognize an Akt1 phosphopeptide.

**Figure 1:**
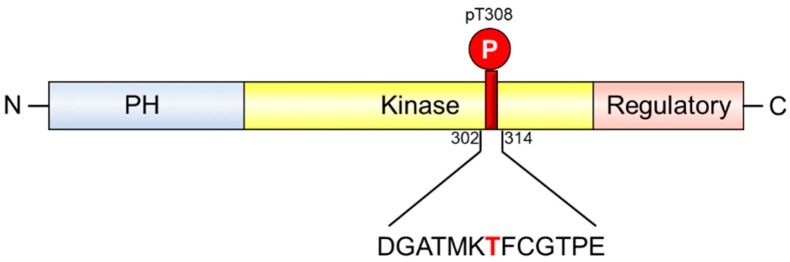
Primary structure of the Akt1 protein. The kinase consists of an N-terminal pleckstrin homology (PH) domain (blue), a serine/threonine kinase catalytic domain (yellow), and a C-terminal regulatory domain (pink). The amino acid sequence from residues 301 to 314, including pT308, was used in peptide form for affinity selection experiments.

**Figure 2:**
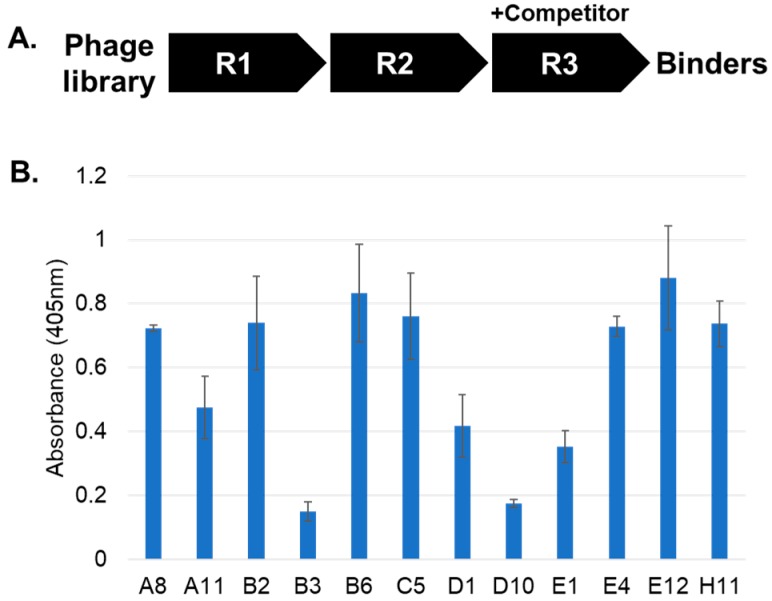
Affinity selection process and ELISA of 12 output clones. (**A**). An M13 bacteriophage library, displaying variants of a thermostable Forkhead-associated 1 (FHA1) H domain, was screened through three rounds (R1–R3) of affinity selection. During R3, excess phosphopeptide was added for off-rate competition, and recovered clones were examined for binding (**B**). Biotinylated Akt1-pT308 peptide was captured on a neutravidin-coated plate and probed with FHA variants that were detected by an M2-HRP conjugated antibody. Error bars represent standard deviation of triplicate measurements.

To generate recombinant affinity reagents that recognize Akt1-pT308, a peptide with a phosphothreonine (pT) residue at position 308 in a 13-mer peptide (positions 302 to 314) was synthesized with a C-terminal biotin (Figure 1). A phage-display FHA library, containing 2 × 10^9^ members [20], was affinity selected with the Akt1-pT308 peptide. After three rounds of affinity selection, 96 recovered clones were analyzed by ELISA and 12 were confirmed to bind to the Akt1-pT308 peptide (Figure 2). None of the 12 binding clones demonstrated any binding to the non-phosphorylated form of the 302–314 Akt1 peptide. 

**Figure 3:**
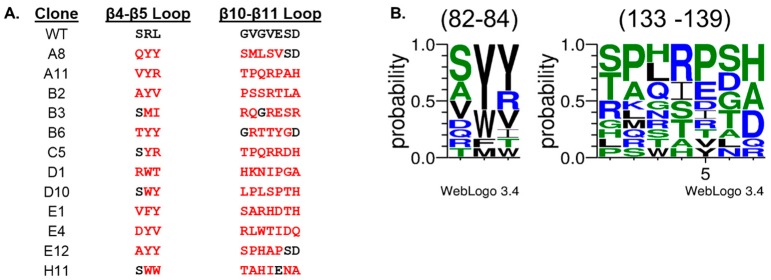
Amino acid sequence analysis of two loops randomized in the phage-displayed scaffold. (**A**) Primary sequences of the wild-type (WT) form of the scaffold and 12 variants that bind the Akt1 phosphopeptide. Three and seven residues in the β4-β5 or β10-β11 loops, respectively, were randomized with NNK codons, where N is an equimolar mixture of A, C, G and T and K is an equimolar mixture of G and T. Residues that differ from the wild-type sequences are show in red. (**B**) WebLogo plots of the frequency of particular residues at each position (82–84 or 133–139). The height of a residue refers to probability of the residue at the given position. Hydrophobic, polar, and charged residues are shown in black, green, and blue, respectively.

DNA sequencing of 12 selected FHA domains revealed that all were unique. A comparison of their coding sequences demonstrated that many of them shared amino acid sequences in the two regions that were randomized in the FHA domain scaffold (Figure 3A). The consensus motif in the β4-β5 and β10-β11 loops determined by a WebLogo plot [24,25] is (S/A)Y(Y/R) and (S/T)(P/A)x(R/I)(P/E)(S/D)(H/A), respectively (Figure 3B). We infer from this finding that the semiconserved positions contribute to binding of the Akt1-pT308 peptide. Correlated with this observation, clones E12 and H11 closely resemble the consensus sequence, and are the strongest binders, whereas clones E1 and B3 are more diverged and are the weakest binders. Among the residues shared most often among the binders is tyrosine at position 83, consistent with previous conclusions that position 83 is extremely important for FHA domain interactions with their phosphopeptide targets [23,26,27]. 

**Figure 4:**
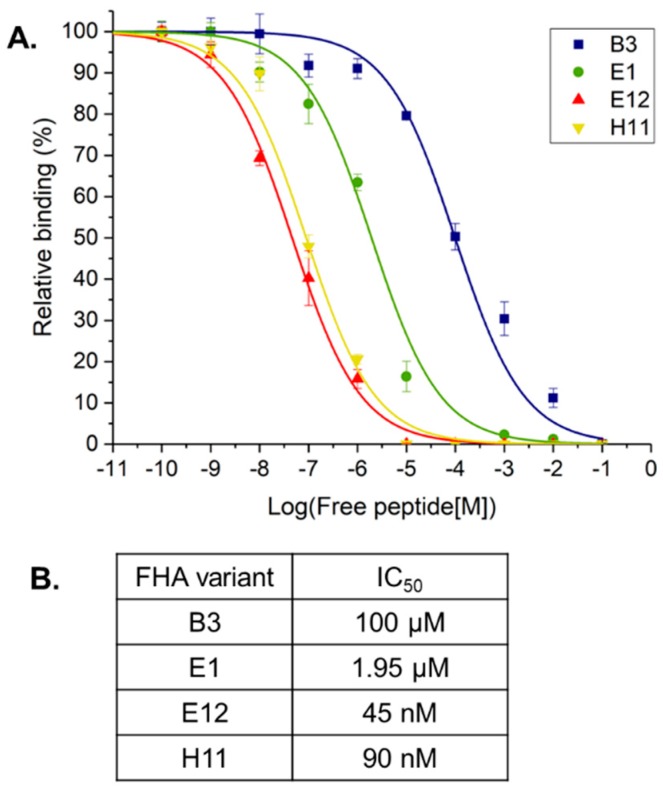
Comparing the relative affinity of four clones. (**A**) Competition binding of clone B3, E1, H11 and E12 to immobilized phosphorylated Akt1 peptide in the presence of free phosphorylated peptide. Error bars represent standard deviation of triplicate measurements. (**B**) IC_50_ values for each clone to the Akt1 phosphorylated peptide.

Two of the affinity selected FHA domains bind the Akt1 phosphopeptide with high affinity. To estimate the binding strength of the affinity selected FHA domain clones for the pT308 peptide, we performed competition binding assays. Half maximal inhibitory concentration (IC_50_) values for clones B3, E1, E12 and H11 were 100 μM, 1.95 μM, 45 nM and 90 nM, respectively. (Figure 4A). These relative values correlated well with ELISA results: clone B3 was the weakest, clones E12 and H11 were the strongest binders, and clone E1 was an intermediate binder (Figure 4B). We then performed surface plasmon resonance (SPR) for two clones, E12 and H11, and determined equilibrium dissociation constants (K_D_) of 162 ± 12 nM and 178 ± 8 nM, respectively (Table 1). These clones are among the tightest binders that we have isolated [18]. 

An isolated FHA variant recognizes the Akt1 phosphopeptide with unique specificity.

**Figure 5:**
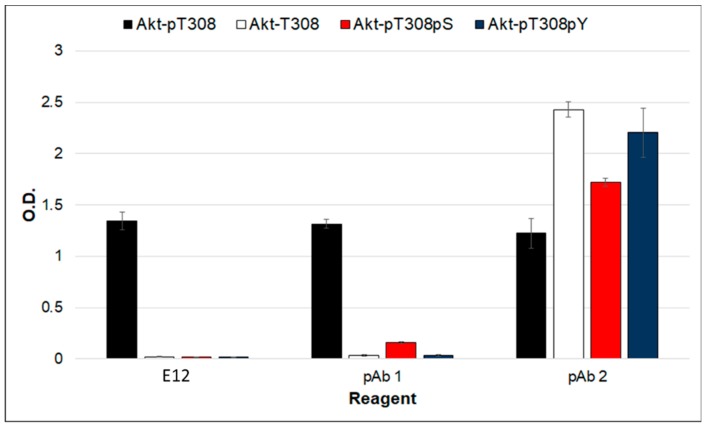
Binding of an Akt1-pTBD to a set of related peptides. A set of biotinylated phosphopeptides containing the Akt1 sequence were synthesized with threonine (Akt-T308), pS (Akt-pS308) or pY (Akt-pY308) substituted at the pT position and immobilized by neutravidin. Binding of the E12 variant and two commercially produced polyclonal antibodies were determined by an ELISA. Error bars represent standard deviation of triplicate measurements.

Due to its unexpected high affinity for its phosphopeptide ligand, we chose to evaluate clone E12 further. The specificity of this FHA variant was examined by testing for binding to peptides that substituted phosphothreonine (pT) in the phosphopeptide with phosphoserine (pS) or phosphotyrosine (pY). As seen in Figure 5, E12 only binds the peptide when position 308 is pT. On the other hand, three commercially-made polyclonal antibodies, which were generated against the same or a similar Akt1 phosphopeptide, varied in their specificity. While one antibody (pAb 1) demonstrated a similar level of selectivity as clone E12, another (pAb 2) lacked specificity, and a third did not bind any of the peptides (data not shown). 

**Figure 6:**
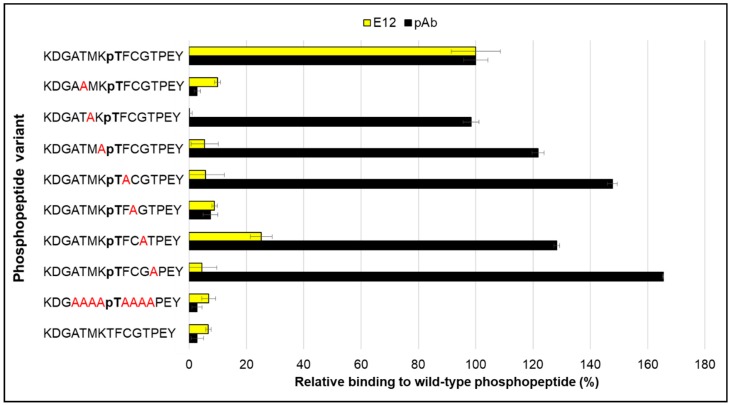
Identification of important residues on the peptide by alanine scanning. Alanine (A) was substituted one at a time at the −3, −2, −1, +1, +2, +3 and +4 positions of the Akt1 peptide sequence. Binding of the E12 variant and a polyclonal antibody (pAb) to the wild type Akt1 peptide target was set to 100%, and the phosphopeptide variants were normalized against it in an ELISA. Error bars represent standard deviation of triplicate measurements.

**Figure 7:**
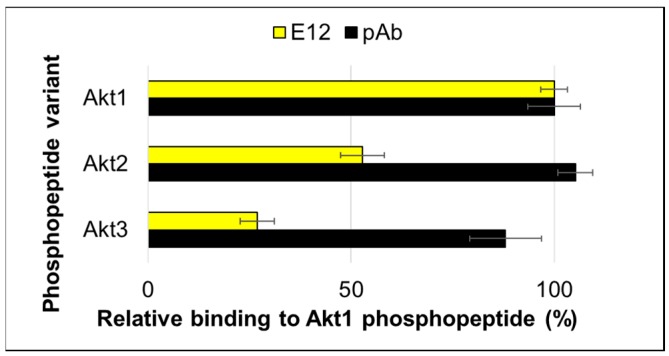
Binding of an Akt1-pTBD to corresponding peptides from Akt2 and Akt3. A set of biotinylated phosphopeptides containing the Akt1 sequence, the Akt2 corresponding sequence (SDGATMKpTFCGTPE) and the Akt3 corresponding sequence (TDAATMKpTFCGTPE) were immobilized by neutravidin. Binding of the E12 variant and a commercially produced polyclonal antibody (pAb) were determined by an ELISA. Error bars represent standard deviation of triplicate measurements.

To learn about what residues in the peptide ligand contributed to binding to clone E12, we tested a set of alanine scanned phosphopeptides. Seven biotinylated peptides, with each position replaced one at a time by alanine, were captured in neutravidin-coated microtiter plate wells and probed separately with a pAb and clone E12 (Figure 6). The pAb bound most alanine-scanned peptides as well as or better than the wild-type sequence, except for alanine replacement at positions −3 and +2 in the phosphopeptide sequence. (By convention the phosphorylated amino acid is defined as 0, and resides N-terminal and C-terminal to are are numbered − and +, respectively.). The pAb did not bind, however, to the non-phosphorylated peptide or to a peptide with alanines flanking the central pT position. Conversely, the E12 clone demonstrated very little or no binding to all of the test peptides. Additionally, it had a reduced ability to bind corresponding peptides from isoforms Akt2 or Akt3 whose sequences differ by only one or two amino acids in positions not tested by the alanine scan (Figure 7). This was surprising since previous observations show that FHA domains recognize only a few nearby residues in addition to the central pT. In this case, though, it appears that many of the residues that flank the central pT in the Akt1 phosphopeptide contribute directly or indirectly to binding. Preliminary molecular dynamic studies of the peptide as well as the solved structure of activated Akt [28] suggest hairpin loops form on both sides of the pT residue, which could explain why so many of the adjacent residues are important directly or indirectly for the binding interaction. While further biophysical experiments are needed to dissect additional details regarding the peptide-FHA domain interaction, the apparent contribution of multiple flanking residues to the interaction correlates with the unusually tight binding observed for the E12 variant and suggests further mutagenesis could increase its specificity for the Akt1 isoform.

Published results demonstrate that the three residues C-terminally adjacent to the pT residue, most notably the +3 residue, contribute significantly to FHA domain interactions with peptides [20,21,23,27]. While many of the 20 amino acids occur among phosphopeptide ligands for FHA domains, the Akt1 pT308 FHA variant is the first one observed to have glycine at the +3 position. Glycine’s small side group and conformational flexibility together may explain why it is not commonly positioned at protein-protein interfaces [29]. Thus, it seems likely that the pTBDs are compensating by interacting more strongly with the remaining peptide residues, and therefore, have a higher specificity and affinity for Akt1 peptide target than other reported FHA binding domains. Given this, clone E12 may serve as an attractive reagent for probing full length phosphorylated Akt1 in western blots, fixed cells, or tissue sections. Such experiments will provide further insight into clone E12’s potential as a diagnostic reagent.

## 3. Materials and Methods

### 3.1. Peptides

Peptides were synthesized at University of Illinois at Chicago’s Research Resource Center with >90% purity. All peptides were biotinylated at their N-terminus and amidated at their C-termini. A phosphopeptide corresponding to human Akt1 was KDGATMKpTFCGTPEY (Akt1-pT308) used as the target in phage display affinity selection. In addition, a number of related peptides were synthesized: KDGATMKTFCGTPEY (T308), KDGATMKpSFCGTPEY (pT308pS), KDGATMKpYFCGTPEY (pT308pY), KDGATMKDFCGTPEY (pT308D), and KDGATMKEFCGTPEY (pT308E), KDGAAMKpTACGTPEY (T305A), KDGATAKpTFCGTPEY (M306A), KDGATMApTFCGTPEY (K307A), KDGATMKpTACGTPEY (F309A), KDGATMKpTFAGTPEY (C310A), KDGATMKpTFCATPEY (G311A), KDGATMKpTFCGAPEY (T312A), and KDGAAAAApTAAAAAPEY (Ala).

Polyclonal antibodies (pAbs) to the Akt1-pT308 peptide were purchased from Abcam (Cambridge, UK), Cell Signaling Technology (Beverly, MA, USA), Millipore (Bedford, MA, USA) and ThermoFisher Scientific (San Jose, CA, USA). The secondary reagent for Akt1-pTBD detection was the anti-Flag epitope mAb, M2, conjugated to horseradish peroxidase (HRP), was purchased from Sigma–Aldrich (St. Louis, MO, USA). The secondary reagent for the pAbs was a goat anti-rabbit immunoglobulin G (IgG), conjugated to HRP (Abcam, Cambridge, UK).

### 3.2. Cloning and Bacterial Expression of Proteins

The phagemid DNA isolated from affinity selection against the Akt1-pT308 peptide were subcloned into the pKP600ΔIII vector [30] and expressed and purified as described elsewhere [23]. Protein purity was assessed by sodium dodecyl sulfate-polyacrylamide gel electrophoresis (SDS-PAGE) and protein concentrations were measured with a NanoDrop A280 spectrophotometer.

### 3.3. Affinity Selections

To isolate FHA variants, the FHA1G2 library [20] was screened against the Akt1-pT308 peptide through three rounds of affinity selection using a modified version of a previously described protocol [20]. All the selection steps were performed at room temperature with the KingFisher™ mL device Purification System (ThermoFisher Scientific, Waltham, MA, USA). The biotinylated peptide (3 ng/μL, 400 μL) was immobilized with Dynabeads™ M-270 Streptavidin (ThermoFisher Scientific, Waltham, MA, USA) and blocked with 2% skim milk in phosphate buffered saline (PBS; 137 mM NaCl, 3 mM KCl, 8 mM Na_2_HPO_4_, 1.5 mM KH_2_PO_4_). A ten-fold excess of the phage library containing 2 × 10^9^ members was incubated with the blocked target for 1 h. Weak or non-binding phage variants were removed by washing the mixture three times with PBS plus 0.1% Tween 20 (PBST) followed by three times with PBS. Virions, which contain a trypsin-cleavable site in the helper M13 viral coat protein III [31], were eluted from the beads with 40 μg of tosyl phenylalanyl chloromethyl ketone-treated trypsin (Sigma-Aldrich, St. Louis, MO, USA ), diluted in 400 μL of 50 mM Tris-HCl (pH 8) and 1 mM CaCl_2_ and used to infect 800 μL of *Escherichia coli* TG1 cells (at mid-logarithmic growth phase) for 1 h at 37 °C. The cells were plated onto one 15 cm petri plates containing 2xYT medium (per liter 16 g tryptone, 10 g yeast extract, 5 g NaCl), 1.4% agar and 0.5 μg/μL carbenicillin, incubated overnight at 37 °C, scraped the next day, and phage amplified. Phage particles were precipitated with 24% polyethylene glycol 8000, 3 M NaCl and the pellet was resuspended in 0.6 mL of PBS. and virions concentrated 30-fold. The second and third rounds of affinity selection were performed in a similar manner; however, the Akt1-pT308 peptide concentration for rounds two and three were reduced to 750 nM and 500 nM in 400 μL of PBS, respectively. Additionally, in the third round of affinity selection, a 10-fold excess of non-biotinylated Akt1-pT308 peptide was added during the wash steps. After the third round of affinity selection, 96 individual clones were propagated as phage and used in an ELISA to identify clones that bind to the peptide target. The DNA inserts of positive, binding clones were sequenced.

### 3.4. ELISA

Biotinylated peptides diluted in PBS were incubated overnight in 0.5 mM DTT at 4 °C. ELISAs were performed as previously described [21,23] using the peptide targets incubated with dithiothreitol (DTT) at a concentration of 2.5 μM in 100 μL and FHA variants at concentrations varying from 0.01 to 10 μM in PBST. The absorbance was read at 405 nanometers (nm) at 10-min intervals, for a total of 40 min. All experiments were performed in triplicate and repeated three times, to confirm reproducibility of the data.

### 3.5. Surface Plasmon Resonance

The affinity of FHA variants E12 and H11 was measured using Biacore T200 following a protocol described elsewhere [32]. The pT308 and T308 biotinylated peptides were diluted to 10 μM with PBS followed by immobilization at each channel with 20 μL/min flow rate for 2 min on the streptavidin (SA) sensor chip, which is coated with streptavidin. A blank channel, without anything immobilized, served as a negative control. The analyte was added in a series of increasing concentration (0.01 to 5 μM) to all four channels at 25 μL/min flow rate for 180 s of dissociation time. 

## 4. Conclusions

Herein, we demonstrate the directed evolution of the FHA domain to bind a phosphorylated peptide that corresponds to a segment of the phosphorylated, oncoprotein, Akt1. The work represented in this paper bolsters the utility of the recombinant FHA domain as pTBD that can serve as an alternative to traditional antibodies for detecting phosphothreonine in peptide sequences. Among the 12 variants isolated from a phage-display FHA domain library, we discovered two that bind the Akt1 phosphopeptide, KDGATMKpTFCGTPEY, with 160–180 nM affinity. This is the strongest interaction between a peptide target and pTBD isolated from our library to date. 

While our FHA variants have yet to be employed as binding reagents against full length protein targets, this FHA probe has the potential to detect phosphorylated Akt1 protein in vivo and/or in vitro due to this high affinity. Thus, we have achieved an early milestone in our goal to replace anti-phosphothreonine antibodies with engineered recombinant pTBDs. Further work to optimize assay detection methods will prove this pTBD’s potential as a detection and diagnostic reagent.

## Figures and Tables

**Table 1:** Affinity measurements of FHA clone E12 and H11 to the phosphorylated (Akt1-pT308) and unphosphorylated (Akt1-T308) peptides.

	FHA Variant					
	E12			H11		
**Peptide Target**	**K_a_ (M^−1^ s^−1^)**	**K_d_ (s^−1^)**	**K_D_ (nM)**	**K_a_ (M^−1^ s^−1^)**	**K_d_ (s^−1^)**	**K_D_ (nM)**
Akt1-pT308	4.830 × 10^4^	7.821 × 10^−3^	162 ± 12	4.157 × 10^4^	7.401 × 10^−3^	178 ± 8
Akt1-T308	2.302	2.144 × 10^−3^	9.31 × 10^5^	2.165	1.0108 × 10^−2^	5.002 × 10^6^
